# Lack of association between revision ACL reconstruction and preoperative, intraoperative and post‐operative factors at primary ACL reconstruction in children and adolescents

**DOI:** 10.1002/ksa.12568

**Published:** 2024-12-19

**Authors:** Riccardo Cristiani, Frida Hansson, Eric Hamrin Senorski, Camilo P. Helito, Kristian Samuelsson, Karl Eriksson

**Affiliations:** ^1^ Department of Molecular Medicine and Surgery Section of Sports Medicine, Karolinska Institutet Stockholm Sweden; ^2^ Stockholm Sports Trauma Research Center (SSTRC), FIFA Medical Centre of Excellence Stockholm Sweden; ^3^ Department of Health and Rehabilitation, Institute of Neuroscience and Physiology, Sahlgrenska Academy University of Gothenburg Gothenburg Sweden; ^4^ Grupo de Joelho, Instituto de Ortopedia e Traumatologia, Hospital das Clínicas HCFMUSP, Faculdade de Medicina da Universidade de São Paulo São Paulo São Paulo Brazil; ^5^ Hospital Sírio Libanês São Paulo São Paulo Brazil; ^6^ Department of Orthopaedics, Institute of Clinical Sciences, The Sahlgrenska Academy University of Gothenburg Gothenburg Sweden; ^7^ Department of Orthopaedics Stockholm South Hospital, Karolinska Institutet Stockholm Sweden

**Keywords:** ACL, anterior cruciate ligament, children, revision ACL reconstruction, revision surgery

## Abstract

**Purpose:**

To evaluate factors associated with revision anterior cruciate ligament reconstruction (ACLR) within 5 years of primary ACLR in children and adolescents.

**Methods:**

Children and adolescents (age <20 years at surgery) who underwent primary hamstring tendon ACLR at the Capio Artro Clinic, Stockholm, Sweden, between January 2005 and December 2018 were identified. Revision ACLR within 5 years of primary ACLR was captured in the Swedish National Knee Ligament Registry. Univariable and multivariable logistic regression analyses were used to evaluate the associations between revision ACLR and preoperative (age, sex, body mass index, time from injury to surgery, pre‐injury Tegner activity level, medial collateral ligament injury, passive contralateral knee hyperextension [≤−5°]), intraoperative (medial meniscus and lateral meniscus [LM] resection or repair, cartilage injury and graft diameter) and post‐operative (KT‐1000 side‐to‐side anterior knee laxity, limb symmetry index for extension and flexion strength and single‐leg‐hop (SLH) test performance at 6 months) factors at primary ACLR.

**Results:**

A total of 1888 patients (mean age: 16.0 ± 2.0, range: 8–19 years) who underwent primary ACLR were included. The overall incidence of revision ACLR within 5 years was 9.0% (170 out of 1888). Univariable analysis revealed that a time from injury to primary ACLR of <5 months (odds ratio [OR]: 2.27, 95% confidence interval [CI]: 1.61–2.35, *p* < 0.001) and LM resection (OR: 1.49, 95% CI: 1.00–2.20, *p* = 0.04) increased the odds of revision ACLR. Multivariable analysis showed that revision ACLR was significantly associated only with a time from injury to primary ACLR of <5 months (OR: 2.56, 95% CI: 1.72–3.70, *p* < 0.001).

**Conclusion:**

There was a lack of association between revision ACLR and preoperative, intraoperative and post‐operative factors at primary ACLR in children and adolescents. A time from injury to primary ACLR of <5 months was the only factor associated with revision ACLR within 5 years.

**Level of Evidence:**

Level III.

**Abbreviations**
ACL: anterior cruciate ligament
ACLR: anterior cruciate ligament reconstruction
BMI: body mass index
HT: hamstring tendon
LM: lateral meniscus
LSI: limb symmetry index
MCL: medial collateral ligament
MM: medial meniscus
ROM: range of motion
SLH: single‐leg‐hop
SNKLR: Swedish National Knee Ligament Registry
STS: side‐to‐side

## INTRODUCTION

The number of children and adolescents undergoing anterior cruciate ligament (ACL) reconstruction (ACLR) has significantly increased in recent years [3, 10]. Although primary ACLR can restore knee laxity and improve subjective knee function [5, 8], graft failure and subsequent revision surgery are common and major concerns in patients after ACLR [1, 7]. Multiple studies have reported that younger age is one of the main factors associated with revision ACLR [2, 7, 19, 22, 34]. However, there is a lack of studies evaluating the factors associated with revision ACLR in children and adolescents.

Revision surgery serves as a significant outcome measure after ACLR. Understanding the factors associated with revision ACLR has important implications for clinicians in terms of providing appropriate patient counselling before primary ACLR. Moreover, knowledge of potentially modifiable factors associated with revision ACLR may be useful in reducing the risk of this serious complication. Therefore, there is a need for a comprehensive analysis of the factors at primary ACLR that are associated with revision ACLR in children and adolescents.

This study aimed to perform a detailed analysis of the preoperative, intraoperative and post‐operative factors at primary ACLR that are associated with revision ACLR within a 5‐year follow‐up period in a large cohort of children and adolescents. It was hypothesised that a shorter time from injury to primary ACLR, high (≥6) pre‐injury Tegner activity level, physiological preoperative contralateral knee hyperextension (≤−5°), symmetrical (limb symmetry index [LSI] of ≥90%) isokinetic extension strength and abnormal anterior knee laxity (side‐to‐side [STS] difference >5 mm) 6 months after primary ACLR would be associated with revision ACLR within 5 years of follow‐up.

## MATERIALS AND METHODS

### Participants

Ethical approval for this study was obtained from the regional ethics committee, Karolinska Institutet (Dnr 2016/1613‐31/2).

The Strengthening the Reporting of Observational Studies in Epidemiology (STROBE) statement was used as guidance to report this study to increase scientific quality [11].

Children and adolescents (age <20 years at surgery) with no concomitant posterior cruciate ligament and/or lateral collateral ligament injuries who underwent primary ACLR using a hamstring tendon (HT) autograft at Capio Artro Clinic, Stockholm, Sweden between 1 January 2005 and 31 December 2018, were included. Patients who underwent contralateral ACLR only contributed with their index knee for analysis.

### Surgical technique and rehabilitation

All patients underwent a transphyseal ACLR using a single‐bundle autologous HT technique. A tripled or quadrupled semitendinosus tendon or a semitendinosus and gracilis tendons were used. An anteromedial portal technique was used to drill the femoral tunnel. The graft was fixed using an EndoButton (Smith & Nephew) or Tightrope (Arthrex) fixation device on the femoral side and Ethibond no. 2 sutures (Ethicon) tied over an AO bicortical screw with a washer as a post. An arthroscopic all‐inside technique using Fast‐Fix suture anchor devices (Smith & Nephew) was used for the repair of meniscal tears located in the posterior horn and body of the menisci, whereas an outside‐in technique using no. 0 PDS (Ethicon) was used for tears located in the anterior horn of the menisci. None of the patients included underwent a lateral extra‐articular procedure (lateral extra‐articular tenodesis or anterolateral ligament reconstruction).

The post‐operative rehabilitation protocol was standardised. Full weight bearing and full range of motion (ROM) were encouraged in the event of an isolated ACLR or an ACLR with concomitant meniscal resection. A hinged knee brace for 6 weeks (0–30° first and second weeks, 0–60° third and fourth weeks and 0–90° fifth and sixth weeks) was used if meniscal repair was performed. From the seventh week, the brace was discontinued, and full ROM was allowed. During the first 3 months, quadriceps strengthening was restricted to closed kinetic chain exercises. Return to sport was allowed 6 months after surgery at the earliest based on knee extension and flexion strength (LSI ≥ 90%) and functional performance (SLH test LSI ≥ 90%) [6, 39].

### ROM and arthrometric evaluation

A goniometer was used to assess passive knee ROM. Knee hyperextension was assessed with the patient's heel placed on a bolster. The goniometer was centred over the lateral femoral condyle with the arms aligned over the lateral midline of the femur (with the greater trochanter as reference) and the lateral midline of the fibula (with the lateral malleolus as reference) [26]. For the purposes of the present study, passive contralateral knee extension was used to avoid the effects of the ACL injury on the injured knee.

Instrumented laxity was assessed 6 months post‐operatively using the KT‐1000 arthrometer (MEDmetric). A standard 30‐lb force (134 N) at 20° of knee flexion was applied. At least three measurements of each knee were performed, and the median value was registered. STS laxity was dichotomised in ≤5 and >5 mm [16].

All measurements (ROM and KT‐1000) were performed by experienced sports medicine physical therapists at our outpatient clinic.

### Isokinetic knee muscle strength and SLH test

Knee muscle strength and the SLH test were assessed 6 months post‐operatively. Isokinetic concentric extension and flexion strength were measured bilaterally at 90°/s using the Biodex System 3 (Biodex Medical Systems). The test was always started with the contralateral knee and performed in a ROM of 90–10°. Before the test, patients warmed up using a stationary cycling ergometer for about 10 min. A verbal description of the test was given, and two or three practical trials were allowed before the test. Five maximum extension and flexion repetitions were performed bilaterally, and the peak torque was registered.

For the SLH test [29], the patient was asked to stay on one leg, jump straight ahead as far as possible and land on the same leg. The test always started with the contralateral leg and was considered successful only if the landing was stable. In the event of loss of balance, additional hops after landing or early touchdown of the contralateral leg, the test was repeated. A verbal description of the test was given, and several practical trials were allowed. Three trials for each leg were performed and the best trial was registered [6, 7, 8].

### Data sources

Data were collected from our clinic database at the Capio Artro Clinic in Stockholm, Sweden. Preoperative factors included age at surgery, sex, body mass index (BMI), time from injury to primary ACLR, pre‐injury Tegner activity level [37], the presence of a medial collateral ligament (MCL) injury and preoperative passive contralateral knee extension. Age was dichotomised into unbiased classes close to the median (≤16 or >16 years). BMI was dichotomised at 25 as patients with a BMI of ≥25 are considered overweight [40]. Time from injury to primary ACLR was also dichotomised into unbiased classes close to the median (<5 or ≥5 months). Knee hyperextension was defined as extension ≤−5° [14]. Intraoperative factors included medial meniscus (MM) or lateral meniscus (LM) resection or repair, the presence of a cartilage injury, and graft diameter. Post‐operative factors (6 months) included were KT‐1000 arthrometric measurements, isokinetic extension and flexion strength, and SLH test performance. An STS laxity >5 mm was considered abnormal (IKDC Grades C and D). Isokinetic knee extension and flexion strength and SLH test performance were considered symmetrical if the LSI was ≥90% [6, 39].

### Revision ACLR

Patients who underwent revision ACLR within 5 years of primary ACLR at our institution or other institutions in the country from 1 January 2005 to 31 December 2023 were identified in the Swedish National Knee Ligament Registry (SNKLR) [38] through their Swedish personal identity number [20].

### Statistical analysis

The Statistical Package for Social Sciences, SPSS (Version 25.0, IBM Corp.), was used for statistical analysis. All the variables were summarised with standard descriptive statistics such as frequency, mean and standard deviation. Univariable analyses were performed with age (≤16 years vs. >16 years), sex, BMI (<25 vs. ≥25), time from injury to primary ACLR (<5 months vs. ≥5 months), pre‐injury Tegner activity level (high ≥6 vs. low <6), MCL injury, contralateral passive knee hyperextension (≤−5°), MM resection, MM repair, LM resection, LM repair, cartilage injury, graft diameter (0.5 mm increase), 6‐month KT‐1000 STS laxity (>5 mm vs. ≤5 mm) and extension and flexion strength and SLH test performance (LSI ≥ 90% vs. LSI < 90%) as independent variables and revision ACLR as the dependent variable. A multivariable logistic regression analysis, including variables attaining a *p *< 0.2 in the univariable analysis, was performed. The results were reported as odds ratios (OR) and 95% confidence intervals (CIs). The level of significance in all analyses was 5% (two‐tailed).

## RESULTS

A total of 1888 children and adolescents who underwent primary ACLR were included. Of these, 170 (9.0%) underwent revision ACLR within the 5‐year follow‐up period. The patient characteristics in the no revision ACLR (*n* = 1718) and revision ACLR (*n* = 170) groups are presented in Table 1.

**Table 1:** Patient characteristics and factors associated with revision ACLR in univariable regression analyses.

	No revision ACLR (*n* = 1718)	Revision ACLR (*n* = 170)	OR (95% CI)	*p*
Preoperative factors				
Age at surgery, years, mean ± SD (range)	16.2 ± 2.0 (8–19)	16.3 ± 1.8 (10–19)		
<16	572 (33.3)	61 (35.8)	0.97 (0.71–1.33)	0.87
≥16	1146 (66.7)	109 (64.2)		
Sex				
Female	990 (57.6)	97 (57.1)	0.98 (0.71–1.34)	0.89
Male	728 (42.4)	73 (42.9)		
BMI, kg/m^2^, mean ± SD	23.3 ± 3.9	23.5 ± 2.7		
≥25	296 (22.3)	22 (21.9)	1.05 (0.70–1.56)	0.80
<25	1033 (77.7)	118 (78.1)		
Patients with available data	1329	151		
Time from injury to surgery, months, mean ± SD	8.6 ± 10.8	5.3 ± 8.0		
<5	772 (44.9)	110 (64.7)	2.27 (1.61–3.22)	<0.001*
≥5	946 (55.1)	60 (35.3)		
Patients with available data	1701	169		
Pre‐injury Tegner activity level, median (range)	8 (1‐10)	8 (1‐10)		
High ≥6	1425 (93.1)	144 (94.1)	1.17 (0.57–2.35)	0.66
Low <6	104 (6.9)	9 (5.9)		
Patients with data available	1529	153		
MCL injury	47 (2.7)	3 (1.8)	0.64 (0.19–2.07)	0.45
Contralateral knee extension, mean ± SD	−3.0 ± 3.6	−3.0 ± 3.9		
≤−5°	696 (43.8)	70 (44.3)	1.01 (0.73–1.38)	0.96
Patients with data available	1587	158		
Intraoperative factors				
MM resection	120 (7.0)	11 (6.5)	0.92 (0.48–1.74)	0.80
MM repair	216 (12.6)	15 (8.8)	0.67 (0.39–1.16)	0.16
LM resection	255 (14.8)	35 (20.6)	1.49 (1.00–2.20)	0.04*
LM repair	174 (10.1)	17 (10.0)	0.98 (0.58–1.66)	0.96
Cartilage injury	126 (7.3)	13 (7.6)	1.05 (0.58–1.89)	0.88
Graft diameter, mm, mean ± SD	8.3 ± 0.7	8.2 ± 1.0		
Per 0.5 mm increase			0.99 (0.78–1.24)	0.94
Patients with available data	1342	146		
Post‐operative factors (6 months)				
KT‐1000 STS difference, mm ± SD	2.1 ± 2.2	2.7 ± 2.2		
>5 mm	71 (5.5)	11 (8.3)	1.60 (0.83–3.09)	0.16
≤5 mm	1217 (94.5)	122 (91.7)		
Patients with available data	1288	133		
Isokinetic extension strength LSI, mean ± SD	89.9 ± 14.1	91.5 ± 11.4		
≥90%	754 (50.6)	83 (57.6)	1.33 (0.94–1.88)	0.10
<90%	736 (49.4)	61 (42.4)		
Patients with available data	1490	144		
Isokinetic flexion strength LSI, mean ± SD	91.1 ± 16.3	91.4 ± 11.9		
≥90%	748 (50.3)	80 (55.5)	1.23 (0.87–1.74)	0.23
<90%	739 (49.7)	64 (44.5)		
Patients with available data	1487	144		
Single‐leg‐hop test LSI, mean ± SD	96.0 ± 11.0	96.8 ± 6.8		
≥90%	1037 (77.8)	107 (81.7)	1.25 (0.79–1.99)	0.34
<90%	296 (22.2)	24 (18.3)		
Patients with available data	1333	131		

*Note*: Data are reported as *n* (%), unless otherwise indicated.

Abbreviations: ACLR, anterior cruciate ligament reconstruction; BMI, body mass index; CI, confidence interval; LM, lateral meniscus; LSI, limb symmetry index; MCL, medial collateral ligament; MM, medial meniscus; OR, odds ratio; SD, standard deviation; STS, side‐to‐side.

* Statistically significant.

### Univariable analyses

Univariable analyses revealed that a time from injury to primary ACLR of <5 months (OR: 2.27, 95% CI: 1.61–3.22, *p* < 0.001) and LM resection (OR: 1.49, 95% CI: 1.00–2.20, *p* = 0.04) increased the odds of revision ACLR. No significant association was found between revision ACLR and age, sex, BMI, pre‐injury Tegner activity level, MCL injury, contralateral passive knee hyperextension (≤−5°), MM or LM resection or repair, cartilage injury, graft diameter, 6‐month KT‐1000 STS laxity and extension and flexion strength, or SLH test performance (Table 1).

### Multivariable analysis

Multivariable logistic regression analysis (total patients included: 1617) showed that the only factor associated with revision ACLR was a time from injury to primary ACLR of <5 months (OR: 2.56, 95% CI: 1.72–3.70, *p* < 0.001). No significant association was found between revision ACLR and MM repair, LM resection, KT‐1000 STS difference of >5 mm and isokinetic extension strength (LSI ≥ 90%) (Table 2).

**Table 2:** Factors associated with revision ACLR in multivariable logistic regression analysis.

	Regression coefficient (*ß*)	SE	OR (95% CI)	*p*
Preoperative factors				
Time from injury to surgery <5 months	0.84	0.19	2.56 (1.72–3.70)	<0.001*
Intraoperative factors				
MM repair	−0.19	0.30	0.83 (0.45–1.51)	0.54
LM resection	0.29	0.22	1.33 (0.86–2.08)	0.20
Post‐operative factors				
KT‐1000 STS difference >5 mm	0.44	0.37	1.55 (0.75–3.22)	0.23
Isokinetic extension strength LSI ≥ 90%	0.29	0.18	1.33 (0.94–1.90)	0.11

Abbreviations: ACLR, anterior cruciate ligament reconstruction; CI, confidence interval; LM, lateral meniscus; LSI, limb symmetry index; MM, medial meniscus; OR, odds ratio; SE, standard error; STS, side‐to‐side.

* Statistically significant.

## DISCUSSION

The most important finding of the present study was the lack of an association between revision ACLR and the preoperative, intraoperative and post‐operative factors at primary ACLR in children and adolescents. A time from injury to primary ACLR of <5 months was the only factor associated with revision ACLR within 5 years. Another important finding was the high (9.0%) overall incidence of revision ACLR within 5 years.

In the present cohort of children and adolescents, age (<16 or ≥16 years) was not associated with revision ACLR. Several previous studies have reported an association between younger age and revision ACLR [2, 7, 19, 22, 35]. Age has been suggested as a surrogate factor for activity level and exposure to pivoting activities [7, 22, 30], with an increased risk of reinjury. Moreover, younger patients may be more willing than older patients to undergo revision surgery if they cannot return to their previous activity level [22, 33]. It may be hypothesised that within the age range included in the present study (children and adolescents aged 8–19 years), these differences are not substantial.

In the present study, sex was not associated with revision ACLR. These results are in line with those of previous studies in adult patients [2, 7, 21, 28, 41], which showed that sex per se is not associated with revision ACLR.

The pre‐injury Tegner activity level was not associated with revision ACLR in the present cohort of children and adolescents. In a recent large cohort registry study, Cristiani et al. [7] reported similar results. It has been hypothesised that patients would not maintain the same activity level post‐operatively [7]. After ACLR, patients may not necessarily be exposed to activities with a high risk of ACL injury, as before surgery.

Timing of the primary ACLR has been previously investigated as a factor potentially associated with revision ACLR in adults. Andernord et al. [1] found no correlation between the time from injury to primary ACLR and revision ACLR. In contrast, Snaebjörnsson et al. [34] found that early (within 3 months) primary ACLR was associated with revision ACLR. Similarly, in the present study, time from injury to primary ACLR of <5 months was associated with revision ACLR within 5 years of the primary surgery in children and adolescents. A longer time from injury to primary ACLR may allow patients to adapt to their injured knee, reducing their exposure to activities that could put them at risk of graft failure and revision ACLR [1, 34]. It is also possible that patients undergoing early primary ACLR may have a higher likelihood of undergoing early revision ACLR, which would be captured within the 5‐year follow‐up period. This could introduce bias, making it appear as though delayed ACLR is associated with a lower risk of revision ACLR [1, 7]. Injuries to the MCL are associated with increased forces in the ACL during valgus and/or external tibial rotation [25, 32]. Svantesson et al. [36] found that patients with isolated ACL injuries who underwent ACLR had a lower risk (hazard ratio: 0.61; 95% CI: 0.41–0.89; *p* = 0.0097) of revision ACLR compared to those with ACLR and non‐surgically treated MCL injuries. However, the present study found that MCL injuries were not associated with revision ACLR within 5 years of the primary surgery in children and adolescents. This is possibly due to the small number of patients with MCL injuries in our cohort (2.7% and 1.8% in the no revision ACLR and revision ACLR groups, respectively).

Whether knee hyperextension is a risk factor for revision ACLR is an ongoing debate. In biomechanical studies, Markolf et al. [23, 24] and Jagodzinski et al. [18] observed higher ACL forces during hyperextension. However, the few clinical studies performed on this topic have reported inconsistent findings. Benner et al. [4] found that knee hyperextension was not associated with ACL graft failure after primary ACLR performed with a bone‐patellar tendon‐bone autograft. In contrast, Guimarães et al. [14] found that patients with hyperextension who underwent primary ACLR with an HT autograft had significantly higher failure rates than those without hyperextension (14.7% vs. 2.9%; *p* = 0.005). In the present study, knee hyperextension (≤−5°) was not associated with revision ACLR within 5 years of primary HT ACLR in children and adolescents.

We were unable to find any association between MM or LM resection or repair during primary ACLR and revision ACLR. Only the univariable analysis showed a weak association between LM resection and revision ACLR (*p* = 0.04), which disappeared in the multivariable analysis. Similarly, cartilage injury was not associated with revision ACLR in this cohort of children and adolescents. In a study based on Swedish and Norwegian knee ligament registries, Snaebjörnsson et al. [34] did not find any correlation between the presence of cartilage injury at the time of the primary ACLR and revision ACLR in adults.

Biomechanical studies have shown a correlation between graft diameter and ultimate failure load [15]. However, the results of clinical studies on the association between graft diameter and revision ACLR in adult patients have been inconsistent. Some authors [21, 27] found a correlation between the HT graft diameter and revision ACLR, whereas others did not [1, 17, 31]. Similarly, in our cohort of children and adolescents, we found that the graft diameter was not associated with revision ACLR.

In the present study, there was no association between an STS laxity difference of >5 mm at 6 months after primary ACLR and revision ACLR. Previous studies in adults have reported conflicting results. Cristiani et al. [9] and Fiil et al. [12] found that early (6 months and 1 year, respectively) post‐operative STS laxity of >5 mm was associated with revision ACLR, whereas Goodwillie et al. [13] found no association between STS laxity of >5 mm at 6 months post‐operatively and revision ACLR. To date, owing to the inconsistent findings in the literature, it is not possible to draw any definitive conclusions regarding the association between anterior knee laxity and revision ACLR.

One unanticipated finding was that the 6‐month isokinetic strength (extension and flexion) and SLH test results were not associated with revision ACLR. At our institution, an important discharge criterion to return to pivoting sports is the achievement of symmetrical (LSI ≥ 90%) isokinetic strength 6 months after primary ACLR. Therefore, it was hypothesised that achieving an LSI ≥ 90% in these tests would be associated with revision ACLR, as patients achieving symmetrical knee strength may have returned to pivoting sports earlier, exposing their knees to the risk of graft failure and revision ACLR. In a large cohort of adult patients, Cristiani et al. [7] found that symmetrical (LSI ≥ 90%) extension strength 6 months after primary ACLR was associated with revision ACLR within 2 years of primary surgery. It may be hypothesised that children and adolescents are less compliant with post‐operative recommendations than adult patients and may have returned to pivoting activities even in the event of asymmetrical knee strength (LSI < 90%) 6 months after primary ACLR.

The main strength of the present study was the analysis of a large cohort (1888 patients) which included a large number of patients (*n* = 170) who underwent revision ACLR. This increased the statistical power, made the results generalisable, and allowed for a detailed analysis of several (preoperative, intraoperative and post‐operative) factors potentially associated with revision ACLR. Data on revision ACLR were collected from a national registry covering all orthopaedic clinics in the country. For this reason, we were able to include patients who underwent revision surgery not only at our institution but also at other institutions. Another strength was that surgery and post‐operative assessments were standardised, as they were performed at the same institution. Similarly, rehabilitation and recommendations for returning to pre‐injury activities were standardised. Finally, another strength was the evaluation of the factors associated with revision ACLR, specifically in children and adolescents.

This study had several limitations. The outcome was revision ACLR and not graft failure, as the SNKLR only registered revision surgeries. Although it is reasonable to believe that most children and adolescents with graft failure undergo revision surgery, the true occurrence of graft failure may have been underestimated. In addition, the factors associated with revision ACLR may differ from those associated with graft failure. However, revision ACLR is a relevant end point which encompasses clinically disabling patient concerns, whereas graft failure is generally defined as increased knee laxity and/or imaging or arthroscopic evidence of graft rupture. Another limitation was that we were unable to control for other factors potentially associated with revision ACLR, such as post‐surgery activity level, return to sports, surgeon experience, ACL tunnel location and anatomical factors (e.g., tibial slope and mechanical alignment). This information is not available in the registry.

## CONCLUSION

There is a lack of association between revision ACLR and the preoperative, intraoperative and post‐operative factors at primary ACLR in children and adolescents. A time from injury to primary ACLR of <5 months was the only factor associated with revision ACLR within 5 years.

## AUTHOR CONTRIBUTIONS

**Riccardo Cristiani**: Conceptualisation; data curation; methodology; writing. **Frida Hansson**: Methodology; critical review of the manuscript. **Eric Hamrin Senorski**: Methodology; critical review of the manuscript. **Camilo P. Helito**: Methodology; critical review of the manuscript. **Kristian Samuelsson**: Methodology; critical review of the manuscript. **Karl Eriksson**: Conceptualisation; methodology; critical review of the manuscript. All authors have given final approval for the version to be published.

## CONFLICT OF INTEREST STATEMENT

The authors declare no conflicts of interest.

## ETHICS STATEMENT

Ethical approval for this study was obtained from the regional ethics committee, Karolinska Institutet, Diarenumber 2016/1613‐31/32. No consent to participate is requested for registry studies.

## Data Availability

The data that support the findings of this study are available from the corresponding author (R.C.) upon reasonable request.

## References

[1] Andernord, D. , Björnsson, H. , Petzold, M. , Eriksson, B.I. , Forssblad, M. , Karlsson, J. et al. (2014) Surgical predictors of early revision surgery after anterior cruciate ligament reconstruction: results from the Swedish National Knee Ligament Register on 13,102 patients. The American Journal of Sports Medicine, 42, 1574–1582. Available from: 10.1177/0363546514531396 24778266

[2] Andernord, D. , Desai, N. , Björnsson, H. , Ylander, M. , Karlsson, J. & Samuelsson, K. (2014) Patient predictors of early revision surgery after anterior cruciate ligament reconstruction: a cohort study of 16,930 patients with 2‐year follow‐up. The American Journal of Sports Medicine, 43, 121–127. Available from: 10.1177/0363546514552788 25325560

[3] Beck, N.A. , Lawrence, J.T.R. , Nordin, J.D. , DeFor, T.A. & Tompkins, M. (2017) ACL tears in school‐aged children and adolescents over 20 years. Pediatrics, 139(3), e20161877. Available from: 10.1542/peds.2016-1877. 28228501

[4] Benner, R.W. , Shelbourne, K.D. & Gray, T. (2016) The degree of knee extension does not affect postoperative stability or subsequent graft tear rate after anterior cruciate ligament reconstruction with patellar tendon autograft. The American Journal of Sports Medicine, 44, 844–849. Available from: 10.1177/0363546515623507 26801922

[5] Cristiani, R. , Forssblad, M. , Engström, B. , Edman, G. & Stålman, A. (2018) Risk factors for abnormal anteroposterior knee laxity after primary anterior cruciate ligament reconstruction. Arthroscopy: The Journal of Arthroscopic & Related Surgery, 34, 2478–2484. Available from: 10.1016/j.arthro.2018.03.038 29752059

[6] Cristiani, R. , Mikkelsen, C. , Forssblad, M. , Engström, B. & Stålman, A. (2019) Only one patient out of five achieves symmetrical knee function 6 months after primary anterior cruciate ligament reconstruction. Knee Surgery, Sports Traumatology, Arthroscopy, 27, 3461–3470. Available from: 10.1007/s00167-019-05396-4 PMC6800857 30778627

[7] Cristiani, R. , Forssblad, M. , Edman, G. , Eriksson, K. & Stålman, A. (2021) Age, time from injury to surgery and quadriceps strength affect the risk of revision surgery after primary ACL reconstruction. Knee Surgery, Sports Traumatology, Arthroscopy, 29, 4154–4162. Available from: 10.1007/s00167-021-06517-8 PMC8595184 33661322

[8] Cristiani, R. , Mikkelsen, C. , Edman, G. , Forssblad, M. , Engström, B. & Stålman, A. (2020) Age, gender, quadriceps strength and hop test performance are the most important factors affecting the achievement of a patient‐acceptable symptom state after ACL reconstruction. Knee Surgery, Sports Traumatology, Arthroscopy, 28, 369–380. Available from: 10.1007/s00167-019-05576-2 PMC6994649 31230125

[9] Cristiani, R. , Forssblad, M. , Helito, C.P. , Edman, G. , Eriksson, K. & Stålman, A. (2024) A high grade of postoperative knee laxity is associated with an increased hazard of revision surgery: a cohort study of 4697 patients with primary ACL reconstruction. The American Journal of Sports Medicine, 52, 1937–1943. Available from: 10.1177/03635465241253840 38819091 PMC11264573

[10] Dodwell, E.R. , Lamont, L.E. , Green, D.W. , Pan, T.J. , Marx, R.G. & Lyman, S. (2014) 20 years of pediatric anterior cruciate ligament reconstruction in New York State. The American Journal of Sports Medicine, 42, 675–680. Available from: 10.1177/0363546513518412 24477820

[11] von Elm, E. , Altman, D.G. , Egger, M. , Pocock, S.J. , Gøtzsche, P.C. , Vandenbroucke, J.P. et al. (2008) The Strengthening the Reporting of Observational Studies in Epidemiology (STROBE) statement: guidelines for reporting observational studies. Journal of Clinical Epidemiology, 61, 344–349. Available from: 10.1016/j.jclinepi.2007.11.008 18313558

[12] Fiil, M. , Nielsen, T.G. & Lind, M. (2022) A high level of knee laxity after anterior cruciate ligament reconstruction results in high revision rates. Knee Surgery, Sports Traumatology, Arthroscopy, 30, 3414–3421. Available from: 10.1007/s00167-022-06940-5 35333934

[13] Goodwillie, A.D. , Shah, S.S. , McHugh, M.P. & Nicholas, S.J. (2017) The effect of postoperative KT‐1000 arthrometer score on long‐term outcome after anterior cruciate ligament reconstruction. The American Journal of Sports Medicine, 45, 1522–1528. Available from: 10.1177/0363546517690525 28277739

[14] Guimarães, T.M. , Giglio, P.N. , Sobrado, M.F. , Bonadio, M.B. , Gobbi, R.G. , Pécora, J.R. et al. (2021) Knee hyperextension greater than 5° is a risk factor for failure in ACL reconstruction using hamstring graft. Orthopaedic Journal of Sports Medicine, 9(11), 23259671211056325. Available from: 10.1177/23259671211056325. 34820464 PMC8606942

[15] Hamner, D.L. , Brown, C.H. , Steiner, M.E. , Hecker, A.T. & Hayes, W.C. (1999) Hamstring tendon grafts for reconstruction of the anterior cruciate ligament: biomechanical evaluation of the use of multiple strands and tensioning techniques. The Journal of Bone & Joint Surgery, 81, 549–557. Available from: 10.2106/00004623-199904000-00013 10225801

[16] Hefti, E. , Müller, W. , Jakob, R.P. & Stäubli, H.U. (1993) Evaluation of knee ligament injuries with the IKDC form. Knee Surgery, Sports Traumatology, Arthroscopy, 1(3–4), 226–234. Available from: 10.1007/BF01560215 8536037

[17] Inderhaug, E. , Drogset, J.O. , Lygre, S.H.L. & Gifstad, T. (2020) No effect of graft size or body mass index on risk of revision after ACL reconstruction using hamstrings autograft. Knee Surgery, Sports Traumatology, Arthroscopy, 28, 707–713. Available from: 10.1007/s00167-019-05395-5 30734062

[18] Jagodzinski, M. , Leis, A. , Iselborn, K.W. , Mall, G. , Nerlich, M. & Bosch, U. (2003) Impingement pressure and tension forces of the anterior cruciate ligament. Knee Surgery, Sports Traumatology, Arthroscopy, 11, 85–90. Available from: 10.1007/s00167-003-0352-0 12664200

[19] Lemme, N.J. , Yang, D.S. , Barrow, B. , O'Donnell, R. , Daniels, A.H. & Cruz Jr., A.I. (2021) Risk factors for failure after anterior cruciate ligament reconstruction in a pediatric population: a prediction algorithm. Orthopaedic Journal of Sports Medicine, 9(3), 2325967121991165. Available from: 10.1177/2325967121991165 34250165 PMC8226238

[20] Lunde, A.S. , Lundeborg, S. , Lettenstrom, G.S. , Thygesen, L. & Huebner, J. (1980) The personal number system of Sweden, Norway, Denmark, and Israel. Vital Health Statistics, 1–59. 7445446

[21] Magnussen, R.A. , Lawrence, J.T.R. , West, R.L. , Toth, A.P. , Taylor, D.C. & Garrett, W.E. (2012) Graft size and patient age are predictors of early revision after anterior cruciate ligament reconstruction with hamstring autograft. Arthroscopy: The Journal of Arthroscopic & Related Surgery, 28, 526–531. Available from: 10.1016/j.arthro.2011.11.024 22305299

[22] Maletis, G.B. , Inacio, M.C.S. & Funahashi, T.T. (2015) Risk factors associated with revision and contralateral anterior cruciate ligament reconstructions in the Kaiser Permanente ACLR Registry. The American Journal of Sports Medicine, 43, 641–647. Available from: 10.1177/0363546514561745 25548148

[23] Markolf, K.L. , Gorek, J.F. , Kabo, J.M. & Shapiro, M.S. (1990) Direct measurement of resultant forces in the anterior cruciate ligament. An in vitro study performed with a new experimental technique. The Journal of Bone & Joint Surgery, 72, 557–567. Available from: 10.2106/00004623-199072040-00014 2324143

[24] Markolf, K.L. , Burchfield, D.M. , Shapiro, M.M. , Shepard, M.F. , Finerman, G.A.M. & Slauterbeck, J.L. (1995) Combined knee loading states that generate high anterior cruciate ligament forces. Journal of Orthopaedic Research, 13, 930–935. Available from: 10.1002/jor.1100130618 8544031

[25] Mehl, J.T. , Kia, C. , Murphy, M. , Obopilwe, E. , Cote, M. , Imhoff, F.B. et al. (2019) Posteromedial ligament repair of the knee with suture tape augmentation: a biomechanical study. The American Journal of Sports Medicine, 47, 2952–2959. Available from: 10.1177/0363546519868961 31454261

[26] Norkin, C.C. & White, D.J. (2003) Measurement of joint motion: a guide to goniometry, 4th ed. Philadelphia, PA: FA Davis.

[27] Park, S.Y. , Oh, H. , Park, S. , Lee, J.H. , Lee, S.H. & Yoon, K.H. (2013) Factors predicting hamstring tendon autograft diameters and resulting failure rates after anterior cruciate ligament reconstruction. Knee Surgery, Sports Traumatology, Arthroscopy, 21, 1111–1118. Available from: 10.1007/s00167-012-2085-4 22688502

[28] Persson, A. , Fjeldsgaard, K. , Gjertsen, J.E. , Kjellsen, A.B. , Engebretsen, L. , Hole, R.M. et al. (2014) Increased risk of revision with hamstring tendon grafts compared with patellar tendon grafts after anterior cruciate ligament reconstruction: a study of 12,643 patients from the Norwegian Cruciate Ligament Registry, 2004‐2012. The American Journal of Sports Medicine, 42, 285–291. Available from: 10.1177/0363546513511419 24322979

[29] Reid, A. , Birmingham, T.B. , Stratford, P.W. , Alcock, G.K. & Giffin, J.R. (2007) Hop testing provides a reliable and valid outcome measure during rehabilitation after anterior cruciate ligament reconstruction. Physical Therapy, 87, 337–349. Available from: 10.2522/ptj.20060143 17311886

[30] Sanborn, R.M. , Badger, G.J. , Fleming, B.C. , Kiapour, A.M. , Fadale, P.D. , Hulstyn, M.J. et al. (2023) Preoperative risk factors for subsequent ipsilateral acl revision surgery after an acl restoration procedure. The American Journal of Sports Medicine, 51, 49–57. Available from: 10.1177/03635465221137873 36412922

[31] Sarakatsianos, V. , Cristiani, R. , Edman, G. & Stålman, A. (2024) Diameter of quadrupled semitendinosus autograft in primary anterior cruciate ligament reconstruction did not impact early revision rate or functional outcome in a large cohort of patients. Arthroscopy: The Journal of Arthroscopic & Related Surgery, S0749‐8063(24)00515‐2. In press. Available from: 10.1016/j.arthro.2024.07.018 39069025

[32] Shapiro, M.S. , Markolf, K.L. , Finerman, G.A. & Mitchell, P.W. (1991) The effect of section of the medial collateral ligament on force generated in the anterior cruciate ligament. The Journal of Bone & Joint Surgery, 73, 248–256. Available from: 10.2106/00004623-199173020-00013 1993720

[33] Shelbourne, K.D. , Gray, T. & Haro, M. (2009) Incidence of subsequent injury to either knee within 5 years after anterior cruciate ligament reconstruction with patellar tendon autograft. The American Journal of Sports Medicine, 37, 246–251. Available from: 10.1177/0363546508325665 19109531

[34] Snaebjörnsson, T. , Hamrin Senorski, E. , Svantesson, E. , Westin, O. , Persson, A. , Karlsson, J. et al. (2019) Graft fixation and timing of surgery are predictors of early anterior cruciate ligament revision: a cohort study from the Swedish and Norwegian Knee Ligament Registries based on 18,425 patients. JB JS Open Access, 4, e0037. Available from: 10.2106/JBJS.OA.19.00037 PMC6959909 32043061

[35] Snaebjörnsson, T. , Svantesson, E. , Sundemo, D. , Westin, O. , Sansone, M. , Engebretsen, L. et al. (2019) Young age and high BMI are predictors of early revision surgery after primary anterior cruciate ligament reconstruction: a cohort study from the Swedish and Norwegian Knee Ligament Registries based on 30,747 patients. Knee Surgery, Sports Traumatology, Arthroscopy, 27(11), 3583–3591. Available from: 10.1007/s00167-019-05487-2 PMC6800860 30879108

[36] Svantesson, E. , Hamrin Senorski, E. , Alentorn‐Geli, E. , Westin, O. , Sundemo, D. , Grassi, A. et al. (2019) Increased risk of ACL revision with non‐surgical treatment of a concomitant medial collateral ligament injury: a study on 19,457 patients from the Swedish National Knee Ligament Registry. Knee Surgery, Sports Traumatology, Arthroscopy, 27, 2450–2459. Available from: 10.1007/s00167-018-5237-3 PMC6656795 30374568

[37] Tegner, Y. & Lysholm, J. (1985) Rating systems in the evaluation of knee ligament injuries. Clinical Orthopaedics and Related Research, 198(198), 43–49. 4028566

[38] The Swedish National Knee Ligament Registry . (n.d.) http://www.aclregister.nu

[39] Thomeé, R. , Kaplan, Y. , Kvist, J. , Myklebust, G. , Risberg, M.A. , Theisen, D. et al. (2011) Muscle strength and hop performance criteria prior to return to sports after ACL reconstruction. Knee Surgery, Sports Traumatology, Arthroscopy, 19, 1798–1805. Available from: 10.1007/s00167-011-1669-8 21932078

[40] WHO Consultation on Obesity and World Health Organization . (2000) Obesity: preventing and managing the global epidemic: report of a WHO consultation, World Health Organization Technical Report Series, 894, pp. i–xii. 1–253. 11234459

[41] Yabroudi, M.A. , Björnsson, H. , Lynch, A.D. , Muller, B. , Samuelsson, K. , Tarabichi, M. et al. (2016) Predictors of revision surgery after primary anterior cruciate ligament reconstruction. Orthopaedic Journal of Sports Medicine, 4(9), 2325967116666039. Available from: 10.1177/2325967116666039 27734019 PMC5042292

